# Multidirectional instability of the shoulder – current concept

**DOI:** 10.1186/1758-2555-1-12

**Published:** 2009-06-25

**Authors:** Seung-Ho Kim

**Affiliations:** 1 Madi Hospital, Seoul, Korea

## Abstract

A guest editorial on the multidirectional instability of the shoulder

## Introduction

The multidirectional instability of the shoulder is a complex problem in terms of diagnosis and treatment. Distinct from multidirectional hyperlaxity, multidirectional instability has symptoms related with increased translations in more than one direction. Increased translation by the increased capsular ligamentous laxity is underlying pathology of the posterior and multidirectional instability. This increased capsular laxity can be in-borne or developmental and asymptomatic or minimally symptomatic initially. In this stage, attempted translation does not produce symptoms. Jerk and Kim tests reveal posterior clunk without shoulder pain [1,2].

**Figure 1:**
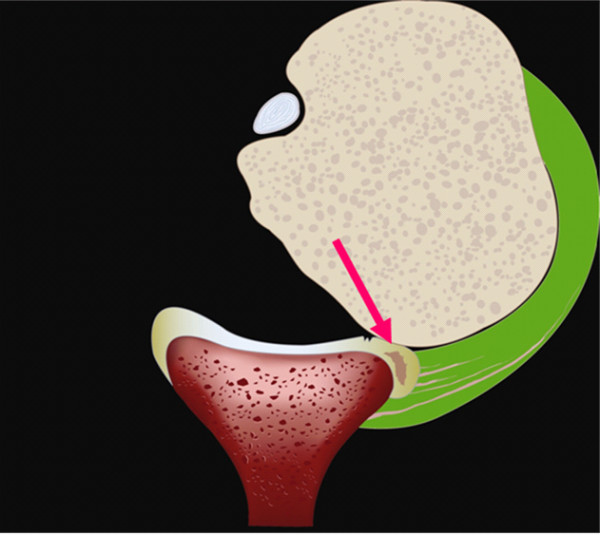
**Excessive rim-loading mechanism**. Repetitive subluxation of the humeral head in the shoulders with hyperlaxity compresses posteroinferior labrum and develops retroversion and eventual fatigue failure of the intra-substance of the labrum which convert the asymptomatic lax shoulder to symptomatic instability.

However, repetitive subluxation overloads the posteroinferior glenoid labrum by the excessive rim-loading of the humeral head. This excessive rim-loading eventually develops posteroinferior labral lesion varying from simple retroversion to incomplete detachment (Figure 1). In this stage, patient's symptom which is shoulder pain, originates from the labral lesion when the humeral head glides over the pathologic labrum. The compressive force on the torn labrum in the jerk and Kim tests generates shoulder pain. Therefore, intact labrum does not produce shoulder pain no matter how lax the glenohumeral joint is. Increased translation alone produces asymptomatic posterior clunk until the repetitive rim-loading eventually develops posteroinferior labral lesion [3].

**Figure 2:**
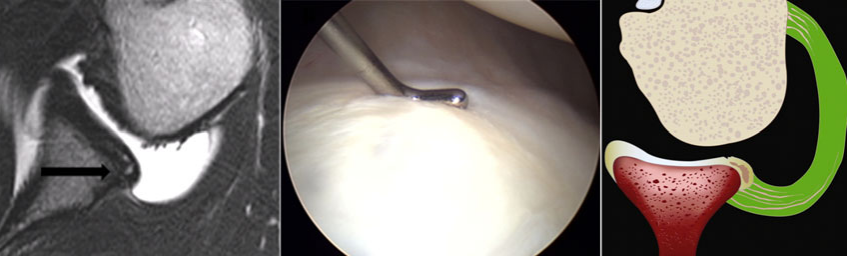
**The Kim lesion**. Arrow indicates a concealed tear in the deep portion of the posteroinferior labrum. Probing reveals soft and loose attachment.

Four type of the labral lesion have been reported. The Kim lesion is a concealed and incomplete tear of the posteroinferior labrum which is characterized by loss of labral height and retroversion, marginal crack, and loose inside. The lesion is similar to the intratendinous tear of the rotator cuff in that it is not evident in the initial observation. The surgeon should be aware of the lesion and palpate with probe (Figure 2).

**Figure 3:**
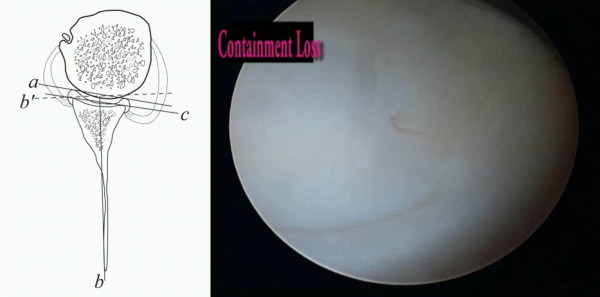
**Loss of containment of the chondrolabral glenohumeral joint**.

The retroversion of the glenoid labrum decrease the containment function of the glenohumeral joint which further decrease the shoulder's stability (Figure 3) [4].

**Figure 4:**
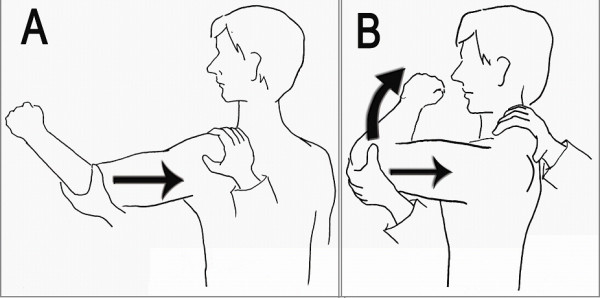
**The jerk test**. Axial loading and horizontal adduction develop posterior clunk with or without pain. Firm axial compression force is important throughout the test.

Two sensitive and specific physical tests are the jerk and Kim tests. Like the McMurray test for evaluation of the meniscal injury in the knee joint, the basic principle of the jerk and Kim tests is a pain provocation by compressing the labral lesion. The jerk test is performed in a sitting position. While stabilizing the patient's scapula with one hand and holding the affected arm at 90-degree abduction and internal rotation, the examiner grasps the elbow and axially loads the humerus in a proximal direction. The arm is moved horizontally across the body. A positive result is indicated by a sudden clunk as the humeral head slides off the back of the glenoid. The painless jerk group includes patients with posterior clunk, but without any significant pain provocation, while the painful jerk group includes patients who show abrupt pain in accordance with posterior clunk [2]. Author found that painful clunk in the jerk test is invariably associated with structural defect, a posteroinferior labral lesion. Majority of shoulders with the painful jerk test fail to improve by the nonoperative treatment. Our arthroscopic finding supported that the abrupt pain during the jerk test may be elicited from a rim-loading of the humeral head over the pathologic posteroinferior labral lesion (Figure 4).

**Figure 5:**
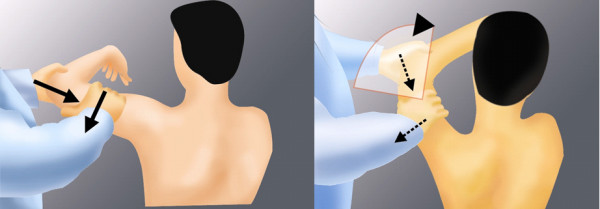
**The Kim test**. Firm axial loading and 45 upward diagonal elevation develops posteroinferior clunk with or without pain.

The Kim test is performed in a sitting position with the arm in 90° abduction. With examiner holding elbow and lateral aspect of the proximal arm, a simultaneous axial loading force and 45° upward diagonal adduction is applied on the distal arm, while downward and backward force is applied on the proximal arm. A sudden onset of posterior shoulder pain indicates positive test regardless of accompanying posterior clunk of the humeral head. During the test, it is important to apply a firm axial force compression force on the glenoid surface by the humeral head [1]. The Kim test was more sensitive in the predominant inferior labral lesion while jerk test was more sensitive for the predominant posterior labral lesion (Figure 5).

**Figure 6:**
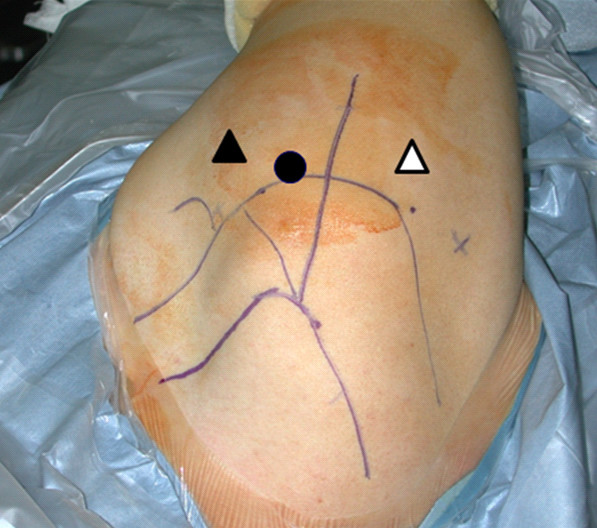
**Triple instability portal**. The black circle indicate transcuff superior portal. The white triangle is the Kim posterior portal and the black triangle is the midglenoid anterior portal.

Initial treatment is supervised rehabilitation including restoration of the scapulothoracic and glenohumeral kinematics. Although strengthening exercises do not decrease hyperlaxity of the shoulder, they improve overall control and function of the shoulder joint. Operative treatment is indicated when nonoperative treatment has failed. The arthroscopic capsulolabroplasty procedure includes posteroinferior labroplasty, superior shift of the posteroinferior and anteroinferior capsule, and posterior portal closure [5,6]. The basic rationale is both restoration of the posteroinferior labral height and capsular tension. Portal placement is critical for successful arthroscopic capsulolabroplasty. We use triple instability portal which includes Kim posterior portal, transcuff superior portal and midglenoid anterior portal. All 3 portals are located vertical from the glenoid surface which allows better access angle to the inferior aspect of glenoid (Figure 6).

## Competing interests

The author declares that they have no competing interests.
